# Atrial Septal Defect vs Ventricular Septal Defect: Getting the Right Mix in Transposition of the Great Arteries

**DOI:** 10.7759/cureus.57518

**Published:** 2024-04-03

**Authors:** Ciaran Cyriac, Thushara Rodrigo, Paolo Hollis, Graham Derrick, Nathalie Dedieu

**Affiliations:** 1 Pediatric Cardiology, Great Ormond Street Hospital, London, GBR

**Keywords:** tga: transposition of the greatarteries, streaming, blood speckle imaging, septostomy, ps:pulmonary stenosis, atrial septal defect (asd), ventricular septal defect (vsd)

## Abstract

Transposition of the great arteries (TGA) is the second most common cyanotic congenital cardiac defect and affects around 4.7 in 10,000 live births. Patients present at birth with profound cyanosis due to inadequate oxygen delivery to the systemic circulation. Typical management after birth involves the administration of prostaglandins and oxygen while awaiting surgical repair. Balloon atrial septostomy may be performed depending on the adequacy of the interatrial communication. In this case report, we present a challenging case of TGA ventricular septal defect (VSD) and pulmonary stenosis (PS), demonstrating the importance of bedside clinical examination along with applying basic management principles. The patient underwent a right modified Blalock-Taussig-Thomas shunt (BTT) along with left pulmonary artery (LPA) reconstruction and main pulmonary artery band as an initial palliative procedure. The patient deteriorated post-operatively, with increasing desaturations and oxygen requirements. Though imaging suggested sufficient inter-circulatory mixing, the clinical picture of desaturation without respiratory distress and lack of response to oxygen and pulmonary vasodilatory therapy strongly suggested otherwise. The child therefore underwent a balloon atrial septostomy. Their clinical condition improved and they were discharged three days later. We describe this case's clinical course, medical and surgical management, and learning points.

## Introduction

Transposition of the great arteries (TGA) refers to the congenital cardiac defect in which there is atrioventricular concordance with ventriculoarterial discordance. The estimated prevalence is approximately 4.7 in 10,000 births [1] and TGA comprises around 5-7% of children with congenital cardiac defects [2]. There is a male preponderance of TGA with a male-to-female ratio of 3:1 [2]. After tetralogy of Fallot, it is the second most common cyanotic congenital cardiac defect [3]. The diagnosis is often made during the antenatal screening processes. Signs of the defect will be evident at or shortly after birth, with cyanosis due to deoxygenated blood from the right heart predominating in the systemic circulation, as well as other signs including dyspnoea, tachypnoea, tachycardia, and poor feeding, depending on associated lesions [4].

In classic complete dextro-TGA (D-TGA) there is separation of the pulmonary and systemic circulations. This defect occurs when the development of the conotruncal septum develops in a linear, rather than spiral, orientation, in utero [1]. Communication between these two parallel circuits is required to ensure the mixing of oxygenated and deoxygenated blood and therefore survival. This mixing can occur through an atrial septal defect (ASD), a ventricular septal defect (VSD), or a patent ductus arteriosus (PDA) [2].

A VSD is present in approximately 30-40% of patients with D-TGA, and a combination of VSD and significant pulmonary stenosis (PS)/left-outflow tract obstruction (as in our case) occurs in approximately 10% of patients with D-TGA [2].

If untreated, the majority of patients will die early and few will survive past two years of age [4]. The normal management process for a TGA involves the administration of prostaglandins after birth to ensure patency of the PDA; administration of oxygen; and urgent transfer to the neonatal cardiac surgical center. Depending on the adequacy of the interatrial communication, a balloon atrial septostomy (also known as Rashkind’s procedure) may be performed before surgery [2]. This involves passing a balloon catheter from the right atrium across the interatrial communication into the left atrium, inflating the balloon, and withdrawing it into the right atrium thereby enlarging the interatrial communication [5].

In simple D-TGA, an arterial switch operation (ASO) is usually performed where the pulmonary trunk and aorta are translocated to the anatomically correct position. The coronary arteries are also translocated [6]. This procedure typically has a good outcome, with one retrospective study of 844 patients who underwent an ASO between 1983 and 2015 identifying an overall survival of 95% at 25 years [7]. However, depending on the other lesions present in addition to transposition, other procedures may be utilized instead. For example in TGA with a VSD and PS, a Rastelli procedure is often used [2]. In this operation, an intracardiac baffle is created tunneling the left ventricle to the aorta and an external conduit connects the right ventricle to the pulmonary artery [8]. A retrospective study of 48 patients who underwent the Rastelli procedure between 1980 and 2017 showed an overall survival of 75.5% at 20 years [9]. A patient with this anatomy typically will require the initial palliative procedure of a Blalock-Taussig-Thomas (BTT) shunt to provide pulmonary blood flow. This allows them to grow to the weight of six to 10 kgs when they can then undergo the Rastelli as a second procedure [8].

In this case report, we describe the management of a neonate with TGA, large inlet to outlet VSD, multilevel PS, restrictive atrial communication, and PDA, who was admitted to our tertiary pediatric cardiology center for further management.

This case was previously presented as a brief conference abstract only at the Eighth World Congress of Pediatric Cardiology and Cardiac Surgery, 2023.

## Case presentation

A male neonate was transferred from a tertiary neonatal unit to our tertiary pediatric cardiology center at nine days of age. They were delivered via emergency cesarean at 38+2 weeks gestation following a failed induction of labor with a pathological cardiotocograph. They were referred due to an antenatal diagnosis, which was postnatally confirmed on echocardiography, of a TGA, large inlet to outlet VSD, multilevel PS, restrictive atrial communication, and PDA. 

On physical examination, heart sounds 1 and 2 were present with an ejection systolic murmur. They were warm and well perfused, with a capillary refill time of < 2 seconds centrally and two to three seconds peripherally. Femoral pulses were palpable and there was no palpable liver edge. The chest was clear with equal air entry. The abdomen was soft and not distended. The anterior fontanelle was soft. 

Due to respiratory distress shortly after birth, the child was intubated and ventilated. On admission to the cardiac intensive care unit, they maintained oxygen saturations above 95% in an FiO2 of 0.21. Prostaglandin was commenced at birth at 5 ng/kg/min which was increased up to 15 ng/kg/min after an echo showed that the PDA was tortuous and restrictive. Full blood count and biochemistry investigations were within normal limits. Pre-surgery ECG showed no abnormalities, with normal sinus rhythm at a rate of 139 beats per minute and right axis deviation. Chest x-ray showed clear lung fields, a mediastinum of normal size, and no pleural effusions.

Following a multidisciplinary team discussion (MDT), it was felt that the patient would ultimately be suitable for a biventricular repair via a Rastelli procedure. However, due to the surgical challenges of a complete repair in a neonate, an initial palliative procedure was undertaken at 14 days of age. This consisted of a right-modified BTT shunt to secure the pulmonary circulation, along with left pulmonary artery (LPA) reconstruction and a main pulmonary artery band. The procedure was uncomplicated with a total bypass time of 62 minutes.

Despite initial improvement in oxygen saturations and complete weaning of respiratory support, the patient subsequently required escalation of respiratory support to continuous positive airway pressure with a FiO2 of 0.8 to maintain saturations over 70%. He showed frequent desaturations with a poor response to oxygen without respiratory distress. A trial of sildenafil, to reduce pulmonary vascular resistance (PVR), was deemed unsuccessful. 

**Figure 1:**
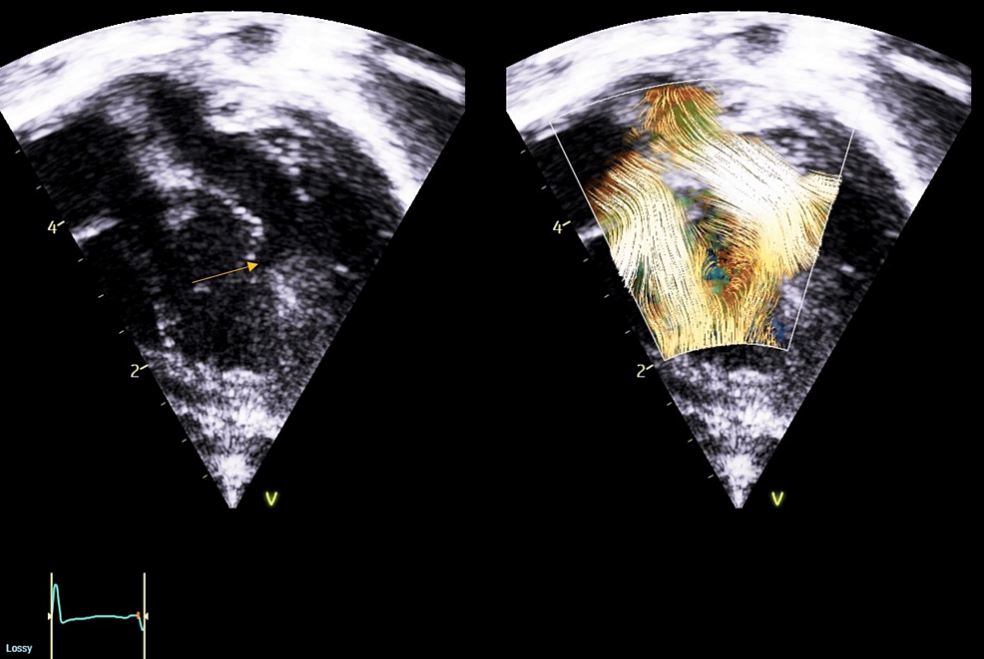
Blood speckle imaging demonstrating mixing across the ventricular septal defect (marked with arrow) (GE Healthcare)

Extensive additional investigations failed to explain the desaturation. Blood tests and chest X-rays were unremarkable. Cardiac CT ruled out any shunt obstruction. An echocardiographic assessment with blood speckle imaging (BSI) suggested satisfactory mixing at ventricular level (Figure 1). However, the clinical picture of desaturation without respiratory distress and lack of response to oxygen and pulmonary vasodilatory therapy strongly indicated poor inter-circulatory mixing. 

**Figure 2:**
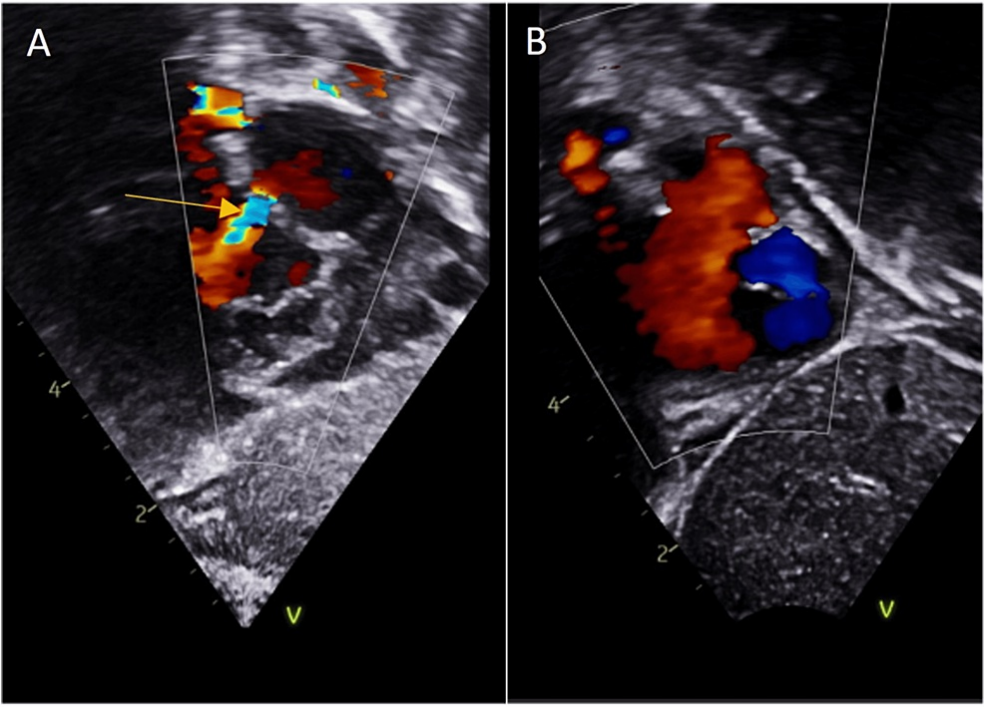
A) Restrictive atrial communication- marked with arrow B) Post-septostomy (GE Healthcare)

Hence, a balloon atrial septostomy (Z5 9.5 mm) was performed following an MDT discussion. The procedure was uncomplicated. Post-procedure echocardiography demonstrated a widely patent atrial communication with a bidirectional flow (Figure 2). The patient was extubated successfully the following day and was gradually weaned off respiratory support over two days and discharged three days later.

## Discussion

It is generally stated that in the presence of a large VSD in TGA, mixing is permitted which can reduce cyanosis and lead to congestive heart failure through pulmonary overcirculation [10]. When left outflow tract obstruction is present and therefore pulmonary blood flow is reduced, the pulmonary vascular bed is protected [2] but cyanosis is increased [10]. However, this is a spectrum that depends on the size of the septal defect, degree of obstruction, and pressure differences between cardiac chambers.

In our case, the infant had significant PS, LPA coarctation, and a large VSD. Although the atrial communication was restrictive, it was felt that once the pulmonary circulation was secured and the antegrade pulmonary blood flow was restricted by a PA band, the VSD would allow efficient intercirculatory mixing and hence maintenance of adequate oxygen saturation. 

Streaming of blood may be a possible mechanism for inadequate mixing across the VSD. This refers to the concept of blood preferentially passing through a common mixing chamber from an inflow to an outflow without complete mixing. For example, in the context of a common ventricle, the pulmonary venous return may preferentially pass to the aorta, which would be favorable streaming. On the contrary, the pulmonary venous return may instead stream to the pulmonary artery, which would be unfavorable. This would lead to lower systemic oxygen saturations despite adequate pulmonary blood flow [11]. This phenomenon has been demonstrated on 4D flow MRI [12] and on catheter studies [13]. 

Previous reports using MRI 4D flow have demonstrated a lack of mixing in this type of circulation [12]. However, this imaging was not available at our center. Due to its non-invasive nature and feasibility, blood speckle imaging (BSI) was performed to assess flow across the VSD in our patient. This is an imaging modality that provides visualization of the speckle pattern originating from blood signal echoes that are not visible on a 2D image due to their weak strength. BSI can demonstrate flow direction independent of the angle of the ultrasound beam and is not limited by velocity aliasing. Therefore, it may provide a greater level of detail compared to color Doppler imaging [14]. BSI has been used to illustrate intracardiac flow in infants with a variety of congenital lesions such as VSDs, ASDs, and PDAs [15]. 

In this patient, no investigations were able to identify the cause of desaturation. Cardiac CT demonstrated adequate flow across the BTT shunt, and BSI appeared to demonstrate adequate intercirculatory mixing across the VSD. It was only the clinical picture of low saturations poorly responsive to oxygen without respiratory distress which pointed towards poor mixing as the likely explanation. During the MDT discussion, ward clinicians were suspicious that imaging was falsely reassuring and hence advocated for balloon atrial septostomy (BAS).

It is possible that while there was some effective systemic blood flow across the VSD as demonstrated by the BSI, there was also unfavorable streaming. Furthermore, while there did appear to flow through the VSD during systole, inter-circulatory mixing is more efficient at an atrial level. This is due to the lower pressure gradient between the chambers, allowing bidirectional flow throughout the cardiac cycle, and therefore allowing more effective pulmonary and systemic blood flow [16]. This may also explain the dramatic improvement following the BAS. 

## Conclusions

This case demonstrates the importance of atrial level mixing to maintain saturations in TGA. Despite having a non-restrictive VSD, BTT shunt, and reassuring advanced imaging, adequate oxygenation was only achieved with atrial septostomy. This highlights that while technology can assist in diagnosis, clinical presentation and applying basic management principles are of paramount importance in guiding management in pediatric cardiology.
